# Modular architecture for fully non-blocking silicon photonic switch fabric

**DOI:** 10.1038/micronano.2016.71

**Published:** 2017-01-16

**Authors:** Dessislava Nikolova, David M. Calhoun, Yang Liu, Sébastien Rumley, Ari Novack, Tom Baehr-Jones, Michael Hochberg, Keren Bergman

**Affiliations:** 1 Department of Electrical Engineering, Columbia University, 530 West 120th Street, New York, NY 10027, USA; 2 Coriant Advanced Technology Group, 171 Madison Avenue, New York, NY 10016, USA

**Keywords:** data communications, optical switching, silicon photonics

## Abstract

Integrated photonics offers the possibility of compact, low energy, bandwidth-dense interconnects for large port count spatial optical switches, facilitating flexible and energy efficient data movement in future data communications systems. To achieve widespread adoption, intimate integration with electronics has to be possible, requiring switch design using standard microelectronic foundry processes and available devices. We report on the feasibility of a switch fabric comprised of ubiquitous silicon photonic building blocks, opening the possibility to combine technologies, and materials towards a new path for switch fabric design. Rather than focus on integrating all devices on a single silicon chip die to achieve large port count optical switching, this work shifts the focus towards innovative packaging and integration schemes. In this work, we demonstrate 1×8 and 8×1 microring-based silicon photonic switch building blocks with software control, providing the feasibility of a full 8×8 architecture composed of silicon photonic building blocks. The proposed switch is fully non-blocking, has path-independent insertion loss, low crosstalk, and is straightforward to control. We further analyze this architecture and compare it with other common switching architectures for varying underlying technologies and radices, showing that the proposed architecture favorably scales to very large port counts when considering both crosstalk and architectural footprint. Separating a switch fabric into functional building blocks via multiple photonic integrated circuits offers the advantage of piece-wise manufacturing, packaging, and assembly, potentially reducing the number of optical I/O and electrical contacts on a single die.

## Introduction

Optical switching has the potential to enable ultra-high capacity optical networks that can deliver large volumes of data with time-of-flight latencies^1–4^. A compact switching device has the potential to enable emerging applications in computing architectures such as optically interconnected processors and memories^5,6^. In the quest to realize scalable optical switch fabrics using various photonic technologies, the primary considerations have focused on individual switching elements and interconnect topologies. A 64×64 port, single chip switch employing micro-electrical–mechanical elements (MEMSs) has been demonstrated^7^; however, operating individual MEMS requires high actuation voltages that are not suitable for all applications. III–V platform, indium phosphide 16×16 switches have been demonstrated^8^, but lack the intimate compatibility with silicon electronic integrated circuits and robust silicon substrates that lower design and fabrication criteria^9^. Silicon photonic crystals also have been studied and demonstrated with compact and low-power switching performance^10^ but require exotic manufacturing schemes. Ultimately, silicon-based optical switching devices benefit from the success of the complementary metal oxide semiconductor (CMOS) industry. Given the maturity of the CMOS process and the easy access to fabrication facilities offered by the fabless model for silicon photonics^11^, silicon photonic technology with standard components offers the most promising platform for practical realization of optical switches.

Small port count silicon photonics switches employing Mach–Zehnder interferometers or microring resonators to provide 1×2 and 2×2 spatial switching have been demonstrated^12,13^ and moreover are readily available to be fabricated using existing foundry services. Hardware–software integrated silicon photonic subsystems controlled by means of custom firmware implemented in field programmable gate arrays (FPGAs)^14–16^ and application-specific integrated circuits (ASICs)^17^ have been demonstrated, offering the possibility for more advanced network functionalities.

Higher port count switches can potentially be realized by cascading multiple 1×2 or 2×2 switching elements. The topology and choice of switching elements determines the on-chip footprint, switch loss, crosstalk impairments, and blocking characteristics. Blocking switches do not guarantee simultaneous transmission from all inputs even if they all have different output port destinations. Reconfigurable non-blocking switches might have to interrupt ongoing transmissions to satisfy an alternative connection requirement^18^, whereas blocking switches simply cannot satisfy some requirements. In a fully (or strictly) non-blocking architecture, the paths from any input port to any output port can be used simultaneously, resulting from the state of the switching elements on a given path being independent from the state of all other switching elements. In addition to overall low losses while propagating through any number of switch elements, path-independent insertion loss (PILOSS)^19^ is desirable for a switch fabric. Fully non-blocking architectures have been realized with crossbar^20,21^, PILOSS^22–24^, and switch-and-select-tree topologies^25^; however, limitations in terms of on-chip footprint, loss, and crosstalk limit the further scalability of such switches^26–29^.

In this work, we propose a silicon photonic system for optical switching that offers to alleviate conventional limitations and is capable of achieving low crosstalk, has weak dependence on port count insertion loss, is fully non-blocking, and has PILOSS. Perhaps, more importantly, this study investigates a switch fabric that leverages the manufacturing advantages of CMOS for innovative system-level design, rather than implementing only exotic materials or device designs to achieve performance. We demonstrate a proof of concept for an 8×8 switch fabric by assembling multiple silicon photonic integrated circuits (PICs) with multiplexing and demultiplexing functionalities. Our analyses show that the proposed architecture can be scaled up to high port counts without significant increase in the insertion loss and crosstalk, even with current state-of-the-art silicon photonic devices. We have implemented software-controlled switching functionality, demonstrating the feasibility of a readily available control plane that can be scaled to even more advanced networking utilities. This easy-to-realize, easy-to-control, and modular architecture can lead to the faster adoption of silicon photonics for optical switches in data communications.

## Materials and methods

### Switch architecture

**Figure 1:**
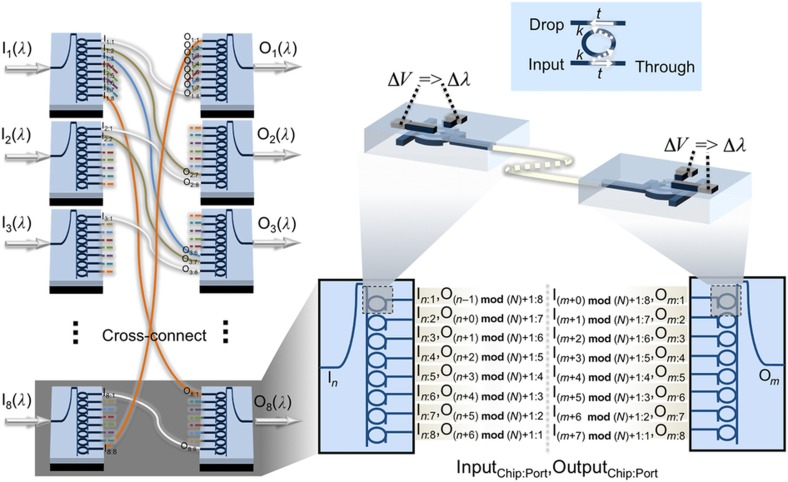
Schematic representation of input–output connectivity of silicon photonic microring switching architecture; (left) depiction of chip-scale integration of eight-microrings multiplexers and demultiplexers to achieve connections between inputs, I_*n*_, and outputs, O_*m*_ (note that not all connections are shown for brevity); (right-bottom) indexed representation of all potential intermediate cross-connects between multiplexers and demultiplexers; (right-middle) implanted on-chip heaters consisting of highly doped regions connected with metallic conductors, inducing a shift in microring resonance proportional to applied power; (right-top) a microring between two waveguides acting as a switch of an input signal between the through and the drop ports.

The proposed switch (Figure 1) consists of multiple silicon PICs interconnected via off-chip cross-connects. Each separate switch input interface is a PIC with a spatial demultiplexer (demux), and each output interface is realized as a PIC with a spatial multiplexer (mux). At each input interface, the optical signal is coupled with the demux PIC acting as a spatial 1-to-*N* switch. At each output interface, optical signals from multiple input interfaces are coupled with one mux PIC acting as a spatial *N*-to-1 switch. Both the demultiplexing and multiplexing functionalities can be realized with *N* microrings coupled with a bus waveguide, as shown in Figure 1. The microrings act as the basic switching element directing an input signal to either the add or drop port (Figure 1, right-top)^30^. One side of the bus waveguide is the ingoing port and the microrings’ drop ports are the chip’s outgoing ports. These microrings can be tuned with an applied voltage to couple light off of the bus waveguide to a drop port. Each microring’s drop port is an outgoing port of the demux chip, and is connected through an off-chip fiber to the ingoing ports of the output interface PIC. Signals are coupled onto the mux PIC via add port waveguides and microrings, and subsequently onto the bus waveguide of the mux and to the mux’s outgoing port. By coupling the signal from the input interface and guiding it to the output interface through optical fibers, the need for on-chip waveguide crossings is mitigated at the cost of chip-to-chip insertion loss. By introducing active switching elements on the output interface via a mux, this architecture aims to significantly reduce inherent architectural constraints of a cross-connect such as crosstalk and subsequent switch scalability.

In this work, we consider only single wavelength switching. As the switch is fully non-blocking, contention can occur only when signals from two or more input ports have to be switched to the same output port. This can be solved by, for example, contention resolution schemes implemented in the control plane. It is worth noting that this architecture is readily applicable to passive and active wavelength routing by tuning the rings to different wavelengths^16^.

The ubiquity of such a microring-based architecture is underlined in the fact that the mux and demux PICs are one and the same, simply used by propagating light in opposite directions.

To ensure that the insertion loss and crosstalk bounds are the same for each input–output pair, the connectivity between the PICs is made such that the signal from any input port to any output port passes through exactly *N*+1 microrings in total. In other terms, the drop port at index *i* must always connect with the add port at index (*N*+1−*i*). The PILOSS connectivity is shown in Figure 1: input demux 1 on outgoing port 1 is connected to ingoing port 8 on output mux 8, and the signal traverses nine total microrings chip to chip. A possible methodology for the assignment of inputs *n* to outputs *m* is also shown in the bottom-right of Figure 1. Although this is not the only method, it can be easily represented in a routing algorithm code implementation as a single and general mathematical function.

### Architectural evaluation

The microrings used for spatial switching in the implemented architecture are especially suited for this type of switch, where the switch elements do not require the same loss to switch the signal from one path to another. Consider a microring between two waveguides (top-right inset in Figure 1): in the ‘bar’ state, the input optical power *P*_in_ propagating along a waveguide will be transferred further along the same waveguide with some loss due to the coupled microring *P*_through_=*T*·*P*_in_. In the ‘cross’ state, part of the power *P*_in_ will couple with the microring and then to the drop waveguide, *P*_drop_=*D*·*P*_in_. Each drop port provides a filtered version of the input signal according to the shape of the resonance, which for a single microring is a Lorentzian.

The through coefficient *T* when the microring is exactly tuned between two resonances and drop coefficient *D* when the ring is on resonance are given with $T=\frac{{\left(1+a\right)}^{2}t^{2}}{{\left(1+t^{2}a\right)}^{2}}$ and $D=\frac{{\left(1-t^{2}\right)}^{2}a}{{\left(1-t^{2}a\right)}^{2}}$, where $a=e^{-\alpha L/2}$ is the optical field loss of a microring with circumference *L* and optical power loss coefficient *α*. The coupling coefficient, *t*, indicates the portion of the incoming electrical field on a waveguide that continues to propagate further along after a microring^31^. *t* depends on the geometry of the coupling region^32^.

The optical signal in the proposed architecture has to be coupled into and out of two chips before being detected on a receiver; subsequently, it must pass through a total of *N*+1 microrings: *N*−1 microrings in the bar state and 2 microrings in the cross state. Furthermore, it has to be guided in an on-chip waveguide, which exhibits a distance-dependent loss *W*_IL_. Taking into account these losses on the optical path, the signal at the output port is given with:

(1)Psignal=PinCIL4WILD2TN−1

where *C*_IL_ is the loss per coupler. The leaked power to any output port *P*_*X*_, is:

(2)PX=Pin CIL4WIL((K−1)(1−T)2TN−1+(N−K)(1−T)2(1−D)TN−2)

where *K* can range from 1 to *N*−1 depending on the destination port of the particular input contributing to the crosstalk. For the case (1−*D*)≤*T*, the maximum total crosstalk power penalty^33^ becomes:

(3a)PPXtalk=−10Log(1−∑i2PX,i/Psignal)

(3b)≤−10Log(1−2(N−1)(1−T)/D)

An upper bound of the total power penalty from the device is given by

(4)PPtotal=ILdB+PPXtalk

For the proposed switch architecture, the insertion loss is given by IL_dB_=−10Log(*P*_signal_/*P*_in_)^33^; *P*_signal_ is given by Equation (1); and the crosstalk power penalty is given by Equation (3a). The loss through a single microring depend on the optical power loss, which is predefined by fabrication specifications, the radius, which also determines the number and position of the resonances and the distance between the microring resonator and the waveguide—which defines coupling—and can be set at device design time^34^. The coupling *t* is therefore the parameter that can be easily optimized to attain lower power penalties. Note that here we have assumed microrings with symmetric coupling but this is not necessary and asymmetric coupling region can be further explored in order to achieve lower power penalties.

**Figure 2:**
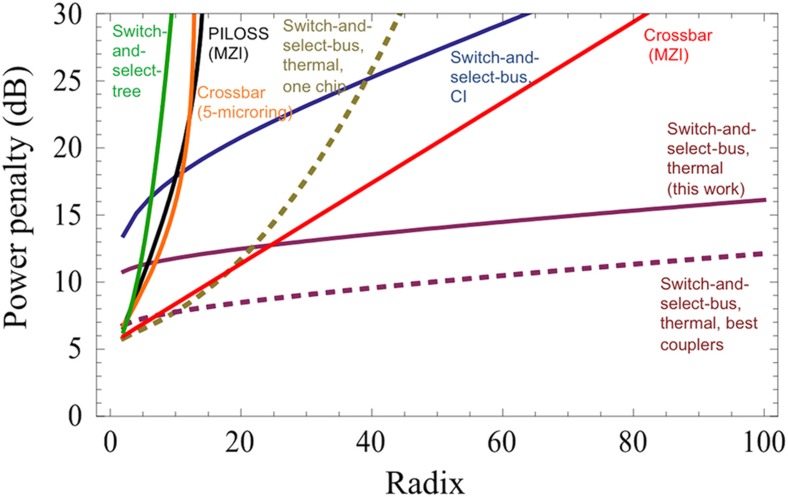
Calculated total power penalty versus switch radix for the proposed systems, three variations of it and other comparable fully non-blocking architectures. The variations are as follows: the entire architecture realized on a single silicon chip, the chip-to-chip architecture realized with carrier injection (CI) tuning, and the chip-to-chip architecture with state-of-the art couplers (best couplers) as recently realized in Ref. 23. The architectures are switch-and-select tree^25^, PILOSS (MZI)^22^, crossbar (five microrings)^21^, and crossbar (MZI)^20^. The power penalty includes losses from the couplers, switching elements, waveguide propagation losses, crossings, and the power penalty due to crosstalk from the switching elements and waveguide crossings. The data are extrapolated based on the data for different on-chip device parameters as reported in the papers that proposed the architecture^21^, as found in foundries (waveguide loss, MZI loss, crossings, and couplers) and for best in class devices (couplers^23^ and waveguide crossing^7^. Full details are given in the Supplementary Information.

Using the optimal coupling value (Supplementary Information S1), the total power penalty was calculated for the proposed architecture according to Equation (4). Figure 2 shows the expected increase in total power penalty according to increasing switch radix. Note that this penalty includes a required excess loss of 10 dB: the sum of losses via four couplers, the waveguide propagation loss, the ‘drop’ loss through two tuned microrings, the ‘through’ loss for passing along *N*−1 detuned rings, and the crosstalk power penalty for *N*−1 interfering signals. All parameters included in this study are currently attainable through existing foundries.

In Figure 2, we also include power penalty results for a selection of comparable silicon photonic switches proposed in the literature, along with the estimated power penalty for three variations of the proposed switch fabric in this work. The notable aspects of variation include the following: the entire architecture realized on a single silicon chip, the chip-to-chip architecture realized with carrier injection tuning, and the chip-to-chip architecture with state-of-the-art couplers as recently realized in Ref. 23. For all architectures, the total power penalty is calculated including insertion losses from fiber/chip couplers, waveguide propagation losses, switch element loss and crosstalk, and on-chip waveguides crossings and crosstalk. The details of the calculations and the included parameters are reflected in Supplementary Information.

Figure 2 clearly shows the high scalability of the proposed switch design in terms of power penalty. Particularly, we see that the design’s high port isolation results in overall low expected power penalty and slow increase according with the switch radix. By avoiding on-chip crossings and optimizing the mode coupling, this switch architecture offers reduced crosstalk due to off-chip cross-connects, making it highly scalable; however, additional interface coupling losses are incurred. Other architectures that do not incur this extra coupling loss suffer from higher crosstalk due to the number of successive switching elements and waveguide crossings in a given path. In the case of the switch-and-select-tree topology, a high number of waveguide crossings scales on the worst case path with (*N*−1)^2^ (Supplementary Table S1). The loss and crosstalk of on-chip waveguide crossings can be significantly improved from the current available foundries values (0.3 dB and approximately −40 dB, respectively). Values of 0.011 dB loss and approximately −70 dB crosstalk have been reported^7^. These best-case values were used to calculate the power penalty for a variation of the proposed switch architecture using all on-chip waveguides similar to the switch-and-select tree^25^. Scaling past a radix of 20, we observe that the loss from the extra two off-chip couplers becomes less than the excess loss from the large number of waveguide crossings required for one chip implementation.

Power penalty scaling for another variation of the proposed architecture, as shown in Figure 2, where carrier injection via p–i–n junctions is used to realize the resonant shift, indicates increased losses in the ring with the required increasing carrier concentration to change the effective index. This increases not only the overall loss but also the crosstalk, which yields higher overall power penalty for this type of switch (Supplementary Information). Such an approach does decrease the required time for switching according to reported speed for electro-optically actuated—specifically, carrier injection—microring modulators^35^, but the inherent trade-off of the switch architecture in terms of power penalty versus switching time is apparent. To further qualify this, we have studied switching times realized with thermally controlled rings, shown in the next section.

The most significant contribution to the total power penalty is from the optical off-chip couplers, which continue to be a focus for improvement in the foreseeable future of optical interfacing. We have shown the expected power penalty for the proposed architecture with each coupler incurring 1.5 dB loss, which was reported in Ref. 23. These results highlight the advantage of the proposed architecture in terms of scalability and will motivate further research into large-scale one- and two-dimensional fiber arrays.

As microrings are resonant devices, they act as filters and subsequently affect modulated data signals^36^. A single microring will incur extra power penalty of ~1 dB for a 10 Gb s^−1^ NRZ signal and ~1.4 dB for a 40 Gb s^−1^ non-return to zero (NRZ) signal. Even though microrings can switch multi-wavelength signals simultaneously, all wavelengths must be aligned with repeating resonances in the microring’s free-spectral range, which is in practice impractical. To switch a wavelength-division-multiplexed signal, the different wavelengths will have to be first filtered out and each has to be switched separately. A filter bank will incur extra power penalty^37^, which has to be accounted for in such multi-wavelength systems.

Scaling to higher port counts requires additional switching elements and therefore a larger footprint. The footprint of a microring is smaller than the one of an Mach-Zehnder interferometer (MZI) switch (Supplementary Table S3), which makes switch fabrics with microrings more scalable in area. In calculating the required chip area, the passive and active photonic device footprint has to be considered, both of which must include the fiber coupling structures^38^. In the case of active photonics, the footprint for electrical controls must also be included in the form of either total number of electrical pads or through-silicon vias^39^, or in-plane electronics components^6^. A rough estimation of the required chip area (Supplementary Information S5) shows that the standard single chip switch designs are limited in scalability not only in terms of power penalty, but also footprint area. This highlights the straightforward challenges in terms of PIC layout and packaging of the proposed design.

## Results

### Device characterization

**Figure 3:**
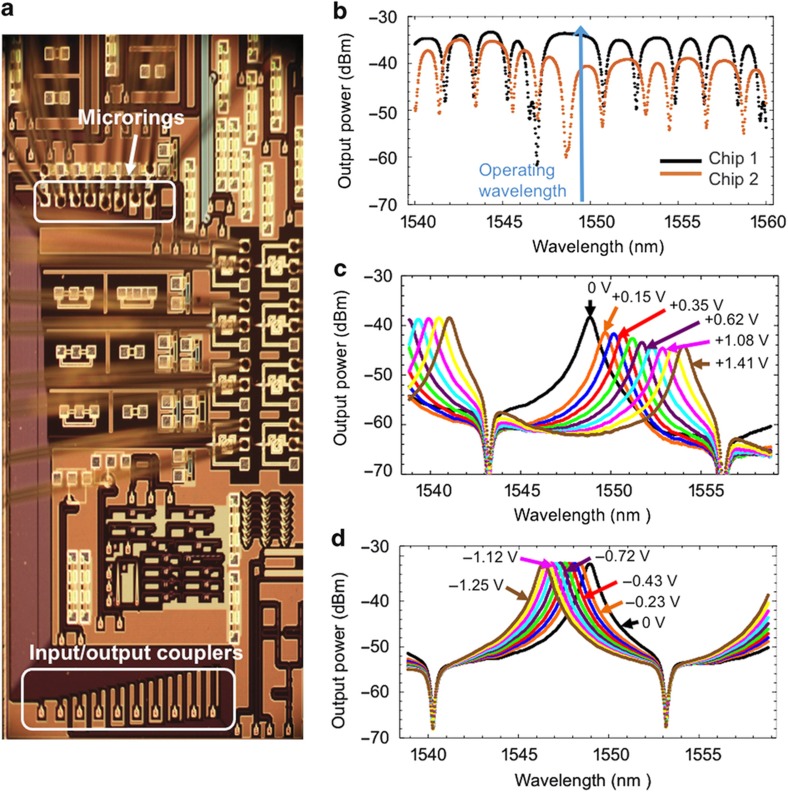
(**a**) An optical micrograph of one wirebonded PIC used in this work. (**b**) The transmission characteristic between the ingoing and outgoing port of the bus waveguides on the two chips. At the operating wavelength, all microrings are off resonance. Tuning the voltage on the thermal heater of a microring (ring 2 in the example) shifts the resonance wavelength and changes the transmitted power to the drop port; (**c**) heating the microring shifts the resonance towards longer wavelength and correspondingly; and (**d**) cooling the microring (decreasing the applied voltage relative to some set initial value) shifts it to shorter wavelengths.

To demonstrate the feasibility of the proposed architecture, we use two eight-channel PICs packaged with electrical wirebonding and epoxy-bonded fiber arrays to grating couplers. A signal being switched through the proposed switch architecture traverses two PICs—at the input and output (I/O) interfaces—and is completely unaffected by the state of the microrings on PICs of other I/O interfaces. The specific PICs used in this work are fabricated at the Institute of Microelectronics/A*STAR, Singapore, via an OpSIS multiple-project-wafer run. The process starts with 8-inch Silicon-on-insulator wafer with 220-nm top silicon layer. Three dry etch steps are used to define the silicon microrings and grating couplers. Six implantation steps are applied to form the heater and contact region in the silicon microrings. Two levels of aluminum are deposited for electrical interconnection. Each eight-channel mux/demux consists of eight silicon microring resonators, side-coupled with a bus waveguide and each coupled with an individual waveguide. The transmission characteristic at the through ports of the bus waveguides of the two chips is shown in Figure 3b. Without any tuning, the ambient resonances of the eight microrings have different frequencies, so the operating wavelength should be such that all ambient microring resonances do not interfere. The free spectral range (FSR) of a microring is ~13.2 nm. The microring is thermally tuned by an integrated heater. The required applied voltage to shift the resonance a full FSR is ~4.2 V. The change of the resonance with the applied voltage for microring 7 on chip 1 (demux) is shown in Figure 3c. Figure 3d shows the same microring on chip 2 (mux). The cascaded effect of the other microrings along the waveguide causes the observed resonance shape to be asymmetric. The microring on chip 1 is heated, whereas the microring on chip 2 is cooled, causing red-shifting and blue-shifting in resonance, respectively. Cooling was achieved by reducing the applied the voltage on the ring relative to its initial value, which was applied to shift its resonance with one FSR. Heating the microring to move it away from resonance results in higher power drop for the same applied voltage than when the microring is cooled due to the asymmetry of the resonance.

### Switching devices and architecture

Three separate experimental approaches were used to demonstrate the efficacy of the chip-to-chip microring cross-connect as a switching architecture with system-level characteristics. These approaches primarily focused on architectural characterization, crosstalk characteristics, and a system-level switching demonstration.

**Figure 4:**
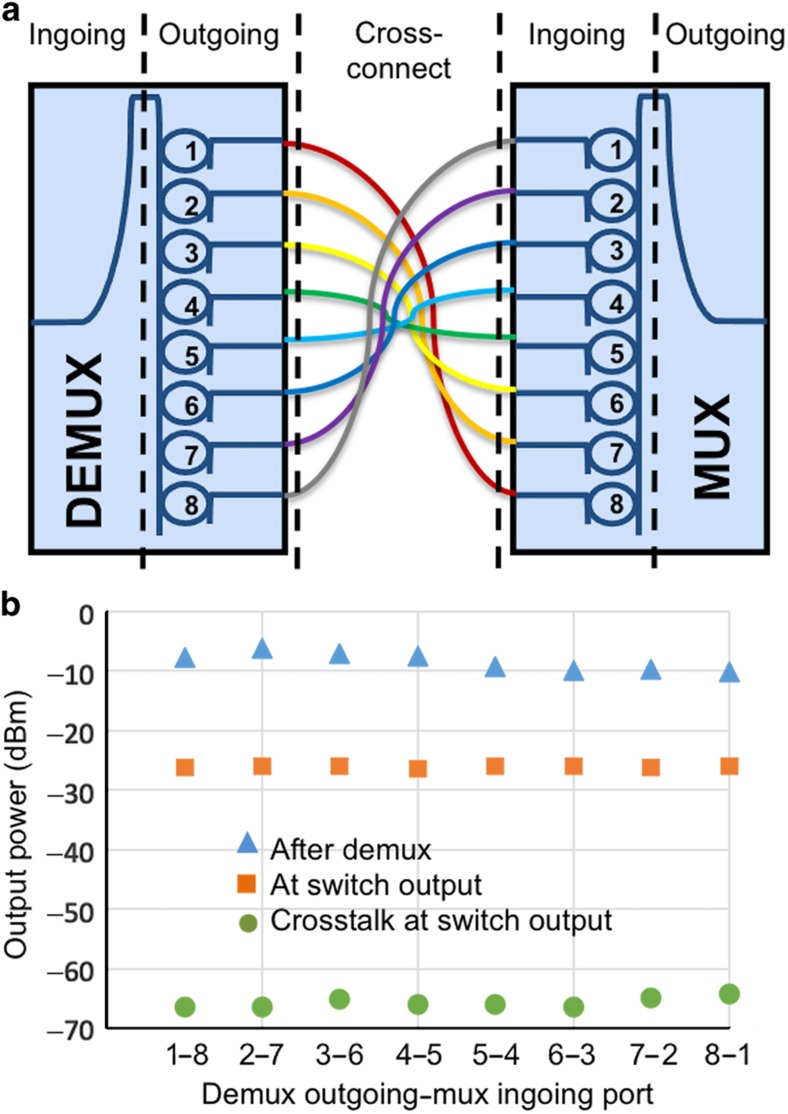
Architectural evaluation scenario utilizing two PICs to emulate the PIC-related insertion loss between the different in- and out-going ports combinations. (**a**) The ingoing–outgoing port configurations with the different colors representing different input–output ports connectivity. (**b**) The measured power of the signal at the outgoing ports of the first chip, after passing the full path through the switch, that is, at the outgoing pot of the second chip and the corresponding crosstalk power when the rings are tuned a half FSR away from resonance. Only one fiber in the cross-connect was coupled for each separate measurement.

The precise connectivity between the ports required to realize an 8×8 switch—ingoing port 1 of demux 1 is connected with ingoing port 8 of mux 1; port 2 of demux 2 is connected with port 7 of mux 1; and so on—is emulated according to Figure 4a. Measuring the insertion loss through the proposed switch is therefore sufficiently demonstrated using only two PICs on separate chip die. Figure 4b shows the output power for the different possible input–output paths between two chips obtained by connecting different ingoing-to-outgoing ports. For each measurement, only one of the connections shown in Figure 4a is realized. The upper row markers show the measured signal power after the first chip on each of its outgoing ports. The polarization of the input signal was set by maximizing the output power from the bus waveguide while passing by all microrings tuned in the bar state. There is a slight decrease in the output power towards higher number of the demux outgoing ports, as the insertion loss increases when the signal is drop from ports situated more towards the end further from the ingoing port. The middle row markers are the measured signal power on the output of the second chip for each ingoing–outgoing port combination as shown in Figure 3a after the signal has passed through seven microrings tuned away from resonance (bar) and two microrings tuned on resonance (cross). The practical insertion loss (~38 dB) of the demonstrated switch fabric is significant due to the employed optical grating coupler technology, inducing ingoing and outgoing insertion losses and mode mismatching^34^. Our previous discussion of fabrication improvements indicates that such loss can be significantly reduced with state-of-the-art coupler designs. Variations in insertion loss on different paths can be attributed to several factors: manufacturing tolerances directly affecting microring size, the geometry of the coupling region between straight waveguides and microrings, and the I/O grating couplers. By tuning the polarization or the applied voltage on the microring, these variations can be minimized and the PILOSS enforced. In our experiment, the polarization of the signal between the paths was tuned such that the output power among the different paths was completely equalized, inducing a scenario where each path experiences the worst-case insertion loss.

The bottom row markers show the measured output power for the different outgoing–ingoing port combinations when the connected microrings are tuned close to half an FSR away from resonance. The power dropped by the microrings while still in the bar state is the crosstalk power each path will contribute to a signal. The polarization of the signal is the same as the polarization that achieves the desired insertion loss. The realized architecture provides excellent port isolation—close to 39 dB.

**Figure 5:**
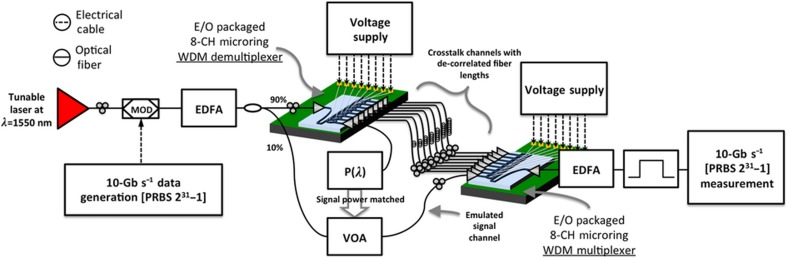
Experimental diagram depicting electrical and optical connections and components used for emulated characterization of the full proposed switching architecture. Voltage supplies used to induce resonance shifts in microrings on the demultiplexer and multiplexer interfaces consisted of digital-to-analog converters controlled by a single FPGA to ensure switching synchronicity. EDFA, erbium-doped fiber amplifier; MOD, modulator.

This low crosstalk power is expected to result in a negligible power penalty to the signal. To demonstrate the crosstalk power penalty, we have measured the bit-error-rates (BERs) of a signal when a varied number of signals contribute to the added crosstalk. To realize this, we utilized the experimental setup as shown in Figure 5. The signal before being coupled with the first chip is split in two as follows: one portion is used to create the crosstalk and the second portion is the measured signal. A passive 90:10 power splitter was used to tap off a copy of the signal generated by modulating a 2^31^–1 pseudorandom sequence on a continuous-wave (CW) laser at 1549.6 nm. This signal was then attenuated and sent to the mux chip where it was dropped by microring 8. The attenuator on its path is used to control its optical power such that it is the same as the power of a signal passing the demux microring 1 to mux microring 8 path. All other microrings except microring 8 on the second chip were tuned away from resonance using an FPGA-enabled tunable voltage supply.

**Figure 6:**
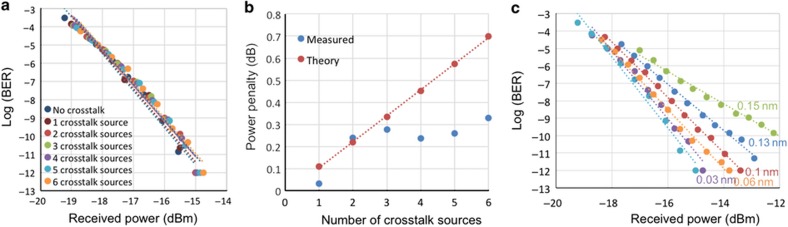
(**a**) The measured BER of the signal without crosstalk and with the number of channels progressively increasing from 1 to 6, adding crosstalk power to the signal. (**b**) The measured power penalty extracted from the BER curves from (**a**) and the corresponding theoretical bound. (**c**) The BER curves for detuned from the resonance microring; the detuning results in lower signal power but the crosstalk power remains the same.

The 90% of the optical power was coupled onto the demux chip to create the crosstalk signals. The different paths emulate six different possible crosstalk paths, consisting of microrings 2 through 7 on the demux connected with 7 through 2 on the mux. All paths are decorrelated using different fiber lengths in the cross-connect. Figure 6b gives the measured and expected power penalty versus the number of crosstalk sources. To calculate an expected value for the power penalty, we obtained the coupling coefficient *t* and optical field loss for each ring from the measured through and drop powers^40^. To obtain *T* for each chip, values for *t* and *a* were taken as the average of these values for rings 2–7. The values for *D* for each chip is obtained from the estimated *t* and *a* for ring 8 for the first chip and ring 1 for the second chip. The theoretical bounds on the power penalty were subsequently obtained from Equation (3b). The measured power penalties were extracted from the BER curves shown in Figure 6a, as the difference between two curves for nominal error-free BER of 10^−12^. The crosstalk powers as indicated in Figure 4b are close to the noise level, and were subsequently observed to fluctuate throughout the experiment. Nevertheless, the theoretical model provides a bound on the measured crosstalk power penalty, which on average remains below the calculated bound. A detuning of the microring resonance will result in less signal power, which will have an effect on the total power penalty. To demonstrate the extent of this effect, the optical attenuator on the signal path was used to measure the BER for different signal powers corresponding to different microring detuning. Figure 6c shows an increase in power penalty according to decreasing signal power, which corresponds to detuning the resonance of one of the two drop microrings on the path from the signal wavelength. The correspondence between detuning and optical power decrease depends on the sharpness of the resonance, which depends on its Q-factor. Microrings with higher Q-factor will be more sensitive to detuning, and methods to control the stability of resonance tuning have been explored^41,42^.

### System-level switching demonstration

We performed an active switching scenario by actuating two microrings on both the input demux and output mux PICs as indicated in Figure 5. A controller circuit board employing an Altera Stratix V FPGA and digital-to-analog converters (DACs) clocked at 65 MHz was used in conjunction with conventional op-amp electrical-level shifters to provide the appropriate voltages to shift each microring’s resonance across its full FSR. This direction-actuation schema to tune microrings to particular resonances consisted of custom firmware—a combination of register transfer level (RTL) hardware description language to configure the DACs and to form an embedded processor—on top of which embedded software code written in C could be executed. This full hardware–software integrated system was used to demonstrate the system-level switching characteristics of the proposed architecture.

**Figure 7:**
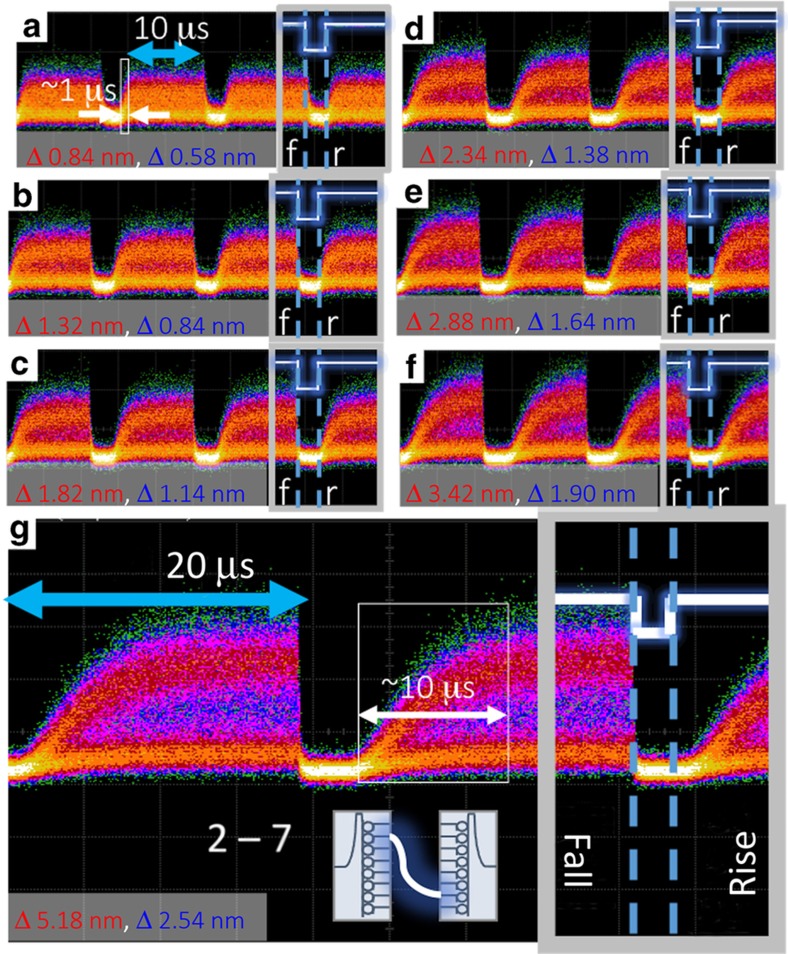
Demux outgoing port 2 to mux ingoing port 7 switching characteristics with respect to red and blue resonance shifts of each microring on each PIC, respectively. (**a**) Demux microring 2 red shifted and mux microring 7 blue shifted of 0.84 and 0.58 nm, respectively. (**b**) Microring 2 red shift 1.32 nm and microring 7 blue shift 0.84 nm. (**c**) Microring 2 red shift 1.82 nm and microring 7 blue shift 1.14 nm. (**d**) Microring 2 red shift 2.34 nm and microring 7 blue shift 1.38 nm. (**e**) Microring 2 red shift 1.88 nm and microring 7 blue shift 1.64 nm. (**f**) Microring 2 red shift 3.42 nm and microring 7 blue shift 1.90 nm. (**g**) Microring 2 red shift 5.18 nm and microring 7 blue shift 2.54 nm. White lines labeled by rise (r) and fall (f) indicate the electrical actuation signal whose associated rise and fall times were on the order of picoseconds, and could not be measured on the same timescale as the optical switching.

The first switching experiment we performed was meant to characterize the rise and fall times of microrings in this switching architecture. We performed an active switching scenario by concurrently actuating two microrings on both the input demux and output mux PICs as indicated in the inset of Figure 7. Figure 7 shows representative results of the entire system: a pair of microring resonances is tuned by fixed amounts across a demux/mux pair. These resonance shifts correspond to a particular voltage sweep, to and from the voltage required to reach resonance tuned to the signal wavelength. A 2^31^–1 pseudorandom binary sequence was modulated onto a CW laser at 1549.6 nm, and was then amplified and injected into the ingoing port of the demux PIC. The signal from the outgoing port of the mux was coupled off-chip, amplified, and observed on a digital communications analyzer.

Although previous works using electro-optic actuation of microring switches show nanosecond and sub-nanosecond rise times^13,35^, the switching set-up time due to thermal actuation in this work is on the order of microseconds. This work shows the relationship of tuning the resonance over varying intervals—or distances shifted between on-resonance and off-resonance—with thermal set-up times at single-digit microseconds without a closed-loop control system. Both red and blue shifts on the demux and mux were used to average the effects of heating and cooling microrings, with the intention of equalizing rise and fall transients. However, the gradual elongation of rise time as observed in Figures 7a–g can be attributed to the fact that when tuning two microrings on resonance, both need to reach resonance before a completed optical path can be constructed. In the case of the fall time, the optical path is deconstructed as soon as either resonance shifts an appreciable amount. We suspect the effect of overall larger resonance shifts on the demux side primarily caused the elongated rise time, the larger resonant shifts for the demux compared with the mux are attributed to the electrical amplification components used level-shift DAC outputs to appropriate voltage swings in this experiment.

The measured rise time for tuning both PICs to establish an optical path in Figure 7a—the small resonance-shift case—is ~1 μs. Figure 7g—the large resonance-shift case—shows an optical path achieved in ~10 μs. The resonance-shift in Figure 7 in between the two extreme cases scale accordingly. The order of magnitude difference between the extremes can be attributed to the rate at which the integrated heaters in the silicon substrate disperse current-induced heat to the microring structure. In the small resonance-shift case, the heat differential between on-resonance and off-resonance microring tuning is smaller than for the large resonance-shift case. In both cases, the rise time of the applied electrical signal is several orders of magnitude faster than the resulting optical rise time, which confirms the limitation on thermal actuation properties primarily influenced by the thermo-optic coefficient of silicon^42^.

**Figure 8:**
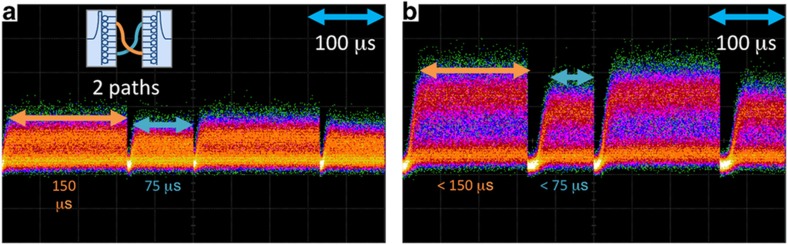
Multipath FPGA-controlled switching on the microsecond scale showing switching between the paths established by demux microring 2 connected with mux microring 7, and by demux microring 2 connected with mux microring 7. (**a**) Switching between two chip-to-chip paths when tuning microring resonances at distances corresponding to Figure 6a; (**b**) switching between two chip-to-chip paths when tuning microring resonances at distances corresponding to Figure 6g. The path holding times are configured for 300 ms on 2–7 and 150 ms on 7–2 in both (**a** and **b**).

We additionally characterized the switching time when transitioning between the two signal paths, as shown in Figure 8. To realize this experiment, we controlled four microrings simultaneously over two chips. We observed similar rise/fall transients, but with different total signal power levels for each channel, likely due to a path differential caused by passive off-chip interconnection components (polarization controllers, fibers, couplers, and so on). This demonstration shows successful multipath switching through the switch architecture, with overall switching times that are suitable for microsecond-scale granularity. In addition, the firmware and software used for switching are easily scalable beyond four microrings due to the modular nature of RTL coding and embedded software coding.

## Discussion

### Impact of active switching

Designing the microrings such that they are off-resonance with the desired wavelengths allows per-port tuning of only two microrings on resonance to achieve port-to-port connectivity. This results in the proposed architecture requiring only two microrings per port to be actively tuned for a switching event at the desired wavelength. In addition, the use of independent switching subsystems at each input and output makes it possible to realize schemes where if the mux/demux at one port is defective, only this port can be replaced or avoided entirely.

In a distributed hardware control architecture, arbitration information is communicated locally to a controller of a 1-to-*N* (equivalent *N*-to-1) switch. We therefore can maintain a single interface for configuration of one 1-to-*N* switch that communicates with a higher-tiered orchestrator, which maintains the status of all 1-to-*N* switches in the full architecture. In this case, the number of interfaces to process control information scales with the number of 1-to-*N* building blocks as 2×*N*.

Control plane scalability is limited by the distribution of controllable objects, that is, the granularity at which we are controlling the full set of 1-to-*N* switches. If we control each 1-to-*N* switch individually and communicate these configurations to a central orchestrator (all implemented with microprocessors or FPGA/ASIC), we effectively distribute the control plane and add complexity and latency in the communications required for control. This control system framework could be tightly integrated—processing chipsets controlling DAC/ADC arrays—all communicating on a central high-speed, addressable bus such as PCI Express. Combining multiple 1-to-*N* PICs on the same chip die conceivably decreases the required number communications interfaces if we assume that the number of interfaces scales directly with the total number of chip die.

A single arbitration controller chipset does not scale for very large optical switch radix when considering connectivity to multiple dedicated analog and digital integrated circuits for control of all microrings. ASICs with analog signaling capabilities, in a multi-chip integration or system-on-chip integration scheme have been demonstrated^43^. Such an integration scheme is a potential solution for controlling a large number of microring devices. Heterogeneous implementation of driver electronics with photonics has been explored at great length, and is a promising solution for application-specific control of photonic elements within the same substrate^44^. Such a solution localizes the optical interconnect and its control to a single substrate, and has the potential for multi-layer photonic–electronic integration.

### Practical considerations

The current market for integrated photonic devices is evolving past innovation on the individual device level and is now moving towards mechanisms for practical interfacing and packaging solutions. We have offered a demonstration of a portion of the full-scale switching architecture via individual 1-to-8 and 8-to-1 microring switches connected using prototypical microelectronic wirebonding and optical grating fiber packaging. Such a packaging solution is not optimal purely considering the cost of the standard classes of optical connectors. Among the possible solutions to realize the cross-connect is a multilayer interposer with passive waveguides and evanescent coupling between the layers^45^. Another interposer technology is a glass interposer with laser imprinted single-mode waveguides^46,47^. This technology permits to alleviate waveguide crossing by varying the *z* coordinate. Resulting waveguides also show excellent propagation properties with the benchmark for propagation loss of 0.05 dB cm^−1^ (Ref. 48). Other packaging solution is photonic wirebonding using polymer waveguides with three-dimensional freeform geometries^49^ or embedded in a printed-circuit board^50^. Nonetheless, the proposed architecture warrants additional innovation in the photonic packaging domain.
