# Supplementary material for: Modular architecture for fully non-blocking silicon photonic switch fabric
Source: Microsyst Nanoeng. 2017 Jan 16;3:16071. doi: 10.1038/micronano.2016.71 (PMC6445003; doi:10.1038/micronano.2016.71)
Supplement: Supplementary Information [file micronano201671-s1.pdf]

Supplementary file

Modular architecture for fully non-blocking silicon photonic switch fabric

Dessislava Nikolova[1,*], David M. Calhoun[1,*], Yang Liu[2], Sébastien Rumley[1], Ari Novack[1,2], Tom Baehr-Jones[2], Michael Hochberg[2] and Keren Bergman[1]

Microsystems & Nanoengineering (2017) **3,** 16071; doi:10.1038/micronano.2016.71; Published online: 16 January 2017

This document provides supplementary material on the optimal coupling coefficient, scaling analysis of selected switching architectures; details of the calculations on the power penalty of using electro-optically controlled rings in the proposed switch architecture, combining multiple muxes(demuxes) on the same chip, and information on the chip area scaling analysis.

S1. INFLUENCE OF THE FIELD COUPLING COEFFICIENT ON THE POWER PENALTY

The dependence on the coupling coefficient *t* of the rings of the total power penalty for the proposed switch, as given by Equation (4) in the main text, is shown on Figure S1. The graph is obtained with parameters $CL_{dB} = 2.5$ dB, $WG_{loss} = 1$ dB cm^{-1} and $a = 0.99$, which are in the standard toolbox in current fabrication facilities (epixfab). We observe that the power penalty remains close to a minimum value over a sufficiently wide range of coupling coefficients, inferring that the proposed architecture is not highly susceptible to fabrication uncertainties. For very large radices, this range becomes smaller and the power penalty will be more sensitive to deviations in the value of optimal coupling. However, the choice of the coupling has large implications on the resulting power penalty and can lead to big gains[1,2].

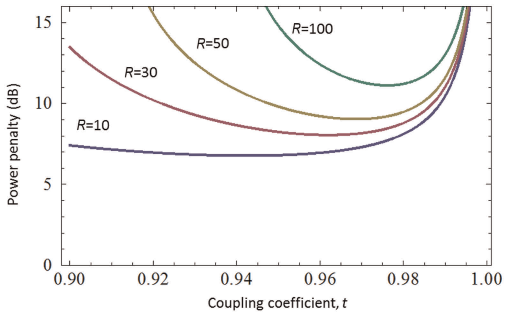

Figure S1 Calculated total power penalty (PP) for the proposed switch design including insertion loss and crosstalk power penalty versus the coupling coefficient *t* for different switch radices (*R*).

S2. ANALYSIS OF THE SCALING OF STATE-OF-THE-ART FULLY NON-BLOCKING SWITCH ARCHITECTURES

Several different switch designs that can be scaled architecturally to high port counts of 8 and more ports have been proposed so far in the literature. They include the PILOSS Figure S2 a), crossbar architectures with switching elements the Mach Zehnder interferometer b) and with microrings c), where the microring switch can be single or multiple coupled, and switch and select d).

For all these architecture the number of needed switching elements is $\sim N^2$. In Table S1 we have summarized the number of passive and active components in the best and worst case optical path these topologies.

The number of waveguide crossings on the optical path differs amongst the architectures but also for the different paths. Waveguide crossings with very low insertion loss have been proposed in the literature, however, considering that for some architectures their number scales with $(N-1)^2$ their loss contribution can be significant. We have also included in the table the number of actively switched elements per port as it gives indication about the energy consumption, where a lower number is desirable.

Besides the single microring switching element considered in the text, other switching elements are the Mach-Zehnder and 5 coupled microrings. The insertion loss in cross and bar state for each device can be made equal with a suitable choice of the device parameters. However, as for some of the architecture the number of elements on the optical path in a given state can different significantly a better design choice would be to design the devices with different loss in the different states. For example in crossbar architecture only one element on the optical path has to be in the drop cross state, while in the worst case the signal has to pass through $2N-2$ elements in the bar state.

An MZI switch can be driven in push-pull state. In the later the insertion loss and cross talk of both cross and bar states are the same. For PILOSS topology this is desirable but for crossbar push-pull state will significantly increase the accumulated crosstalk and loss. In crossbar topology regardless of the switching element only one element along the optical path has to change state to guide the signal. Hence with MZI switches the elements should be used such that when the element is in cross state for which no voltage needs to be applied the signal remains on the path and when voltage is applied to put the switch in bar state the signal changes paths. For example for the path In1->Out3 only the third switch on the first row needs to be in bar state and all others should be in cross state. The big advantage is that for ideal MZI switch the crosstalk in cross state is 0 and the insertion loss is equal to the waveguide propagation loss. In push pull state the insertion loss $IL = ((1+\exp(-\Gamma a L/2)/2)^2$ and the crosstalk defined as the crosstalk

[1]Department of Electrical Engineering, Columbia University, 530 West 120th Street, New York, NY 10027, USA and [2]Coriant Advanced Technology Group, 171 Madison Avenue, New York, NY 10016, USA.
Correspondence: Dessislava Nikolova (dessie.nikolova@gmail.com)

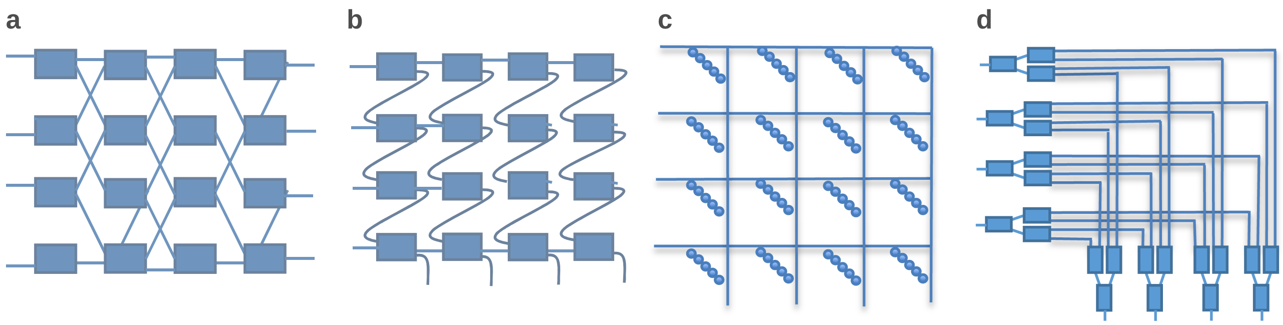

Figure S2 Switch architectures. (**a**) PILOSS; (**b**) crossbar with MZI; (**c**) crossbar with 5-microrings; and (**d**) switch-and-select-tree.

power divided by the signal power is given by XT = $\tanh^2(-\Gamma \alpha L/4)$. $\Gamma = 0.7$ is the mode confinement factor for the MZI switching element and for push pull state $\alpha = 8.35\ \text{cm}^{-1}$ while for single ended $\alpha = 18.65\ \text{cm}^{-1}$. In switch and select architecture the push pull state is preferable as this is double gated architecture and any crosstalk has second and higher order contributions which we consider to be neglectable. Note that XT = $P_{X,i}/P_{\text{signal}}$ as given in Equation (3a) in the main text.

For the microrings crossbar topology there are also crossings on the optical path unlike MZI switching elements. A microrings crossbar switch has been demonstrated with each switching element consisting of 5 coupled microrings. We consider the worst case path to be from In 1 to Out 1 where each other input contributes to the crosstalk at output 1 $P_{X,i} = D_5(\text{OFF}) * T_5^{i\ \ 1}(\text{OFF}) W_{\text{IL}}^{i+1}$ and the signal at output 1 is $P_{\text{signal}} = D_5(\text{ON}) * T_5^{N\ \ 1}(\text{OFF}) W_{\text{IL}}^{N+1}$, where W_{IL}^1 is the waveguide loss along the length of single switching element, D_5 (T_5) is the transmission via the 5-microring switching element in drop (through), which depends whether the ring is ON or OFF resonance. It is likely possible to optimize an on chip crossbar with microring switching elements to have lower total power penalty by optimizing the coupling and size of the rings.

Equation (3a) in the main text represents a conservative upper bound on the crosstalk power penalty independent of the required bit-error-rate (BER) for data links on a switch. It closely approximates the BER dependent crosstalk power penalty as derived in Ref. 1 for low BERs.

S3. SCALING OF CARRIER INJECTION CONTROLLED MICRORING SWITCH

In order to achieve nanoseconds switching times the microrings have to be controlled using the free-career plasma dispersion effect by implementing a p-i-n junction [\cite ns switching time]. To achieve 2 nm shift in the resonance wavelength of the ring the refractive index has to change with $\Delta\lambda/\lambda$ which accounting the Soref's formulas[2]

$$n_{\text{eff}} = \quad \left(8.8 \quad 10^{\ \ 22} \text{d}N + 8.5 \quad 10^{\ \ 18} \text{d}P^{0.8}\right)$$

$$\Delta\alpha = 8.5 \quad 10^{\ \ 18} \text{d}N + 6 \quad 10^{\ \ 18} \text{d}P$$

requires change in the carrier concentration with $\text{d}N \sim 1.87 \times 10^{18}$. This results in $\Delta\alpha \sim 27\ \text{cm}^{-1}$, for a ring with size $r = 7.54\ \mu\text{m}$. This gives for the optical power loss in the ring off resonance $\alpha = 0.99$ and on resonance $\alpha = 0.87$.

A smaller shift will result in smaller losses and a more in-depth trade-offs analysis can lower the power penalty for carrier-injection microring switches.

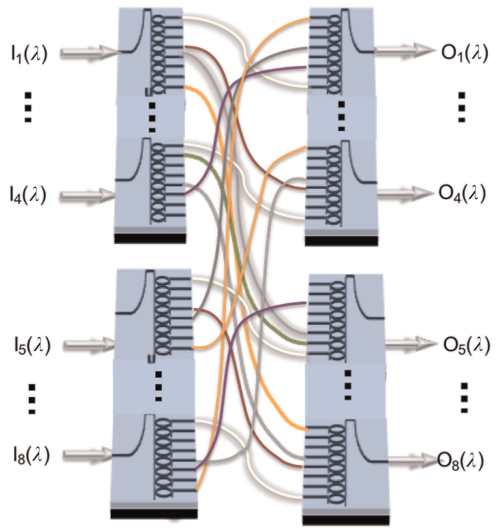

Figure S3 Schematic representation of the proposed switch design combining the muxes (demuxes) of multiple ports on one chip.

S4. COMBINING MULTIPLE MUX(DEMUX) INPUT(OUTPUT) INTERFACES ON ONE CHIP

We note that, instead of having a separate chip for each port, multiple muxes can be laid out on the same chip. The muxes for the separate I/O ports remain completely independent as shown on Figure S3. There the mux/demux of 4 input/output ports are grouped on the same chip. In this way for an 8 port switch instead of having 2 sets of 8 chips (16 total) with only 8 microrings each, we have 2 sets of 2 chips (4 total) with 32 microrings each, grouped as 4 independent muxes.

The cross connect is still off chip, and there are no on-chip crossings. All input and output interfaces are completely independent, but the number of mux/demuxes that can be combined on one chip will depend on the required area for devices, fiber couplers and electrical pads. This shows the flexibility of the proposed architecture and how it can be reduced to an architecture on two chips only, causing any electrical or optical I/O scalability issues to become similar to other single chip architectures.

S5. AREA SCALING ANALYSIS

To estimate the on-chip area required by each switch topology we have used the number of switching elements and crossings given in Table S1 and the components' footprints given in Table S3. To

Table S1 Comparison of switch architectures

	Nr of switching elements	Nr of switching elements on the optical path		Nr of crossings on the optical		Nr of actively switched elements path per port
		Best case	Worst case	Best case	Worst case	
PILOSS (MZI)	N^2	N	N	0	N–1	N
Crossbar (MZI)	N^2	1	2N–1	0	0	1
Crossbar (5-microring)	N^2	1	2N–1	0	2N–1	1
Switch and select-tree (MZI)	2N (N–1)	$2\mathrm{Log}_2N$	$2\mathrm{Log}_2N$	0	$(N-1)^2$	$2\mathrm{Log}_2N$
Switch and select-bus (microring)	$2N^2$	N+1	N+1	0	0	2

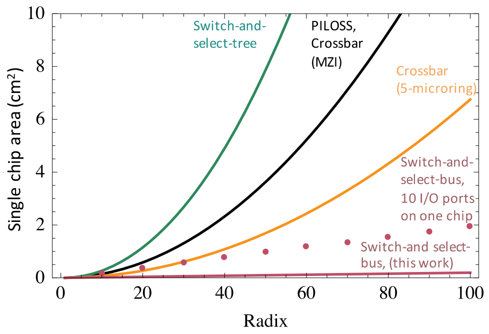

Figure S4 Estimation of the scaling of the required chip area for a single chip from the proposed architecture, for multiple I/O mux/demuxes combined on one chip and other comparable fully non-blocking architectures considering the same area for the Crossbar and PILOSS switches with MZI switching elements.

Table S2 Summary of the losses for passive photonic components

Parameter	Best in class	Typical foundry values
	Value	Value
Fiber/chip coupling loss, dB	1.5	2.5
MMI crossing loss, dB	0.011	0.3
MMI crossing crosstalk, dB	–70	–40
On-chip waveguide loss, dB cm^{-1}	0.25	1

account for the electrical pads we have considered the pad area as 150 × 150 microns multiplied by the number of switching elements for the corresponding design and further multiplied by two as each element needs at least two electrical connections.

The fiber coupler pitch has typical values of 125 um but to be scaled other solutions like 2-dimesionial fiber array need to be used which has pitch of around 40 microns [Ref. 38, main text]. We have neglected this area in the presented results in Figure S4.

Figure S4 shows an estimation of the footprint area for a single PIC with the parameters given in Table S3. The single chip area is important for cost purposes. With separate chips each holding a limited number of rings, the probability of having a defect making the chip unusable is also limited. The required footprint for the proposed in this work switch architecture is small and well within reach of current fabrication methods, and is therefore compatible

Table S3 Summary of the on-chip footprint of typical photonic components

Parameter	Value	Unit
Length MZI cell	400	µm
Widht MZI cell	200	µm
Length microring cell	150	µm
Width microring cell	150	µm
Electrical pad pitch	150	µm
Waveguide crossing area	625	μm^2
Fiber coupler pitch	125 (40)	µm

with both optical and electrical packaging schemes. Additionally, we investigated the possibility of combining multiple input (output) spatial multiplexers on a single chip die in order to reduce the total number of chips needed without altering the architecture as discussed in Section S4. As an example, an estimation of the area for combining 10 input muxes (output demuxes) on one chip is shown on Figure S4. Even for a 100-port switch, each chip will require 1000 switching elements and the required area is at a reasonable 2 cm^2. Note that the total switch will consist of 2 sets of 10 such chips to achieve 100 input and output ports. The number of mux/demux that can be put on a single chip die is limited only by area scaling restrictions because all muxes/demuxes are independent.

Fiber coupling architectures with relatively small footprint that can scale to high port-counts are commercially available, and continue to scale [Ref. 38, main text]. The electrical pad area might be more critical as the perimeter scales with $N^{½}$ while the number of pads scales with N, but several rows of electrical pads can be used and bonded with conventional microelectronic processes such as wirebonding. There is also the option of flip-chip attach [Ref. 39, main text], which is another common microelectronic process used for processor-die. This method continues to be fairly uncommon in photonics currently due to additional post processing or the difficulty in using grating couplers after bonding. We also show the required footprint for other switch designs using single chips and typical values indicated in Table S3.

REFERENCES

1 Takahashi Hiroshi, Oda Kazuhiro, Hiromu Toba. Impact of crosstalk in an arrayed-waveguide multiplexer on N x N optical interconnection. *Journal of Lightwave Technology* 1996; **14**: 1097–1105.

2 Soref R., Bennett B. Electrooptical effects in Silicon. *IEEE Journal of quantum electronics* 1987; **23**: 123–129.
